# The role of milk-derived exosomes in the treatment of diseases

**DOI:** 10.3389/fgene.2022.1009338

**Published:** 2022-10-21

**Authors:** Mehdi Rashidi, Salar Bijari, Amir Hossein Khazaei, Fereshteh Shojaei-Ghahrizjani, Leila Rezakhani

**Affiliations:** ^1^ Department of Medical Nanotechnology, Islamic Azad University of Pharmaceutical Sciences (IAUPS), Tehran, Iran; ^2^ Nano Drug Delivery Research Center, Health Technology Institute, Kermanshah University of Medical Sciences, Kermanshah, Iran; ^3^ Department of Medical Physics, Faculty of Medical Sciences, Tarbiat Modares University, Tehran, Iran; ^4^ Student Research Committee, Shahid Beheshti University of Medical Sciences, Tehran, Iran; ^5^ Department of Pharmacological and Biomolecular Sciences, University of Milan, Milan, Italy; ^6^ Fertility and Infertility Research Center, Health Technology Institute, Kermanshah University of Medical Sciences, Kermanshah, Iran; ^7^ Department of Tissue Engineering, School of Medicine, Kermanshah University of Medical Sciences, Kermanshah, Iran

**Keywords:** milk-derived exosome, clinical applications, exosome isolation, natural nanovesicles, diseases

## Abstract

Exosomes (EXOs) are natural nanoparticles of endosome origin that are secreted by a variety of cells in the body. Exosomes have been found in bio-fluids such as urine, saliva, amniotic fluid, and ascites, among others. Milk is the only commercially available biological liquid containing EXOs. Proof that exosomes are essential for cell-to-cell communication is increasingly being reported. Studies have shown that they migrate from the cell of origin to various bioactive substances, including membrane receptors, proteins, mRNAs, microRNAs, and organelles, or they can stimulate target cells directly through interactions with receptors. Because of the presence of specific proteins, lipids, and RNAs, exosomes act in physiological and pathological conditions *in vivo*. Other salient features of EXOs include their long half-life in the body, no tumorigenesis, low immune response, good biocompatibility, ability to target cells through their surface biomarkers, and capacity to carry macromolecules. EXOs have been introduced to the scientific community as important, efficient, and attractive nanoparticles. They can be extracted from different sources and have the same characteristics as their parents. EXOs present in milk can be separated by size exclusion chromatography, density gradient centrifugation, or (ultra) centrifugation; however, the complex composition of milk that includes casein micelles and milk fat globules makes it necessary to take additional issues into consideration when employing the mentioned techniques with milk. As a rich source of EXOs, milk has unique properties that, in addition to its role as a carrier, promotes its use in treating diseases such as digestive problems, skin ulcers, and cancer, Moreover, EXOs derived from camel milk are reported to reduce the risk of oxidative stress and cancer. Milk-derived exosomes (MDEs) from yak milk improves gastrointestinal tract (GIT) development under hypoxic conditions. Furthermore, yak-MDEs have been suggested to be the best treatment for intestinal epithelial cells (IEC-6 cell line). Because of their availability as well as the non-invasiveness and cost-effectiveness of their preparation, isolates from mammals milk can be excellent resources for studies related to EXOs. These features also make it possible to exploit MDEs in clinical trials. The current study aimed to investigate the therapeutic applications of EXOs isolated from various milk sources.

## Introduction

Newborns must adopt milk as their sole source of nutrition to support themselves so as to grow during the early stages of life (Silva et al., 2020). As a result, milk is much more important than other nutritional sources. To provide this source of nutrition to the public, vast amounts of milk ranging from 708 million to 883 million tonnes were produced worldwide between 2009 and 2019. Not only is milk one of the best sources of nutrition, but it also has many therapeutic applications in medical research (Ong et al., 2021). Extracellular vesicles are particles comprising phospholipid bilayers that are secreted by many cells throughout the body. They are categorized into three subgroups: apoptotic bodies, microvesicles, and EXOs. Apoptotic bodies (1,000–5,000 nm) are derived from apoptosis cells, microvesicles (100–1,000 nm) bud off from plasma membranes, and EXOs (30–150 nm) are endosomal in origin. EXOs are natural extracellular nano-vesicles that contain biologically active substances like proteins, microRNA, mRNA, DNA, and other molecules that play an essential role in interacting with various types of cells (Shirkhanloo et al., 2017; Rahmati et al., 2020; Rashidi et al., 2021).

Surprisingly, EXOs are found in most biological fluids, including plasma, urine, saliva, milk, amniotic and cerebrospinal fluids, etc (Van niel et al., 2018; Sedykh et al., 2020; Fontana et al., 2021). Milk-derived exosomes (MDEs) are among the most important signaling molecules that mediate cellular communication between a mother and her offspring (Sedykh et al., 2020).

Interestingly, EXOs can be effectively isolated from many different types of milk, including bovine (Pieters et al., 2021), Porcine (Chen et al., 2014), Yak (Gao et al., 2019), Camel (Badawy et al., 2018), Human breast (Admyre et al., 2007), Goat (Santos-Coquillat et al., 2022). Isolated EXOs are durable in size and biological activity until stored frozen (−80°C), because they are protected by a phospholipid bilayer, The barrier prevents miRNAs in EXOs from degrading in the gastrointestinal tract and from being absorbed deeper in the gut (Izumi et al., 2012; Van Hese et al., 2020). The current review investigated a wide range of methods used to isolate MDEs, MDE sources, and applications for MDEs.

## Biogenesis and identification of exosomes

**FIGURE 1:**
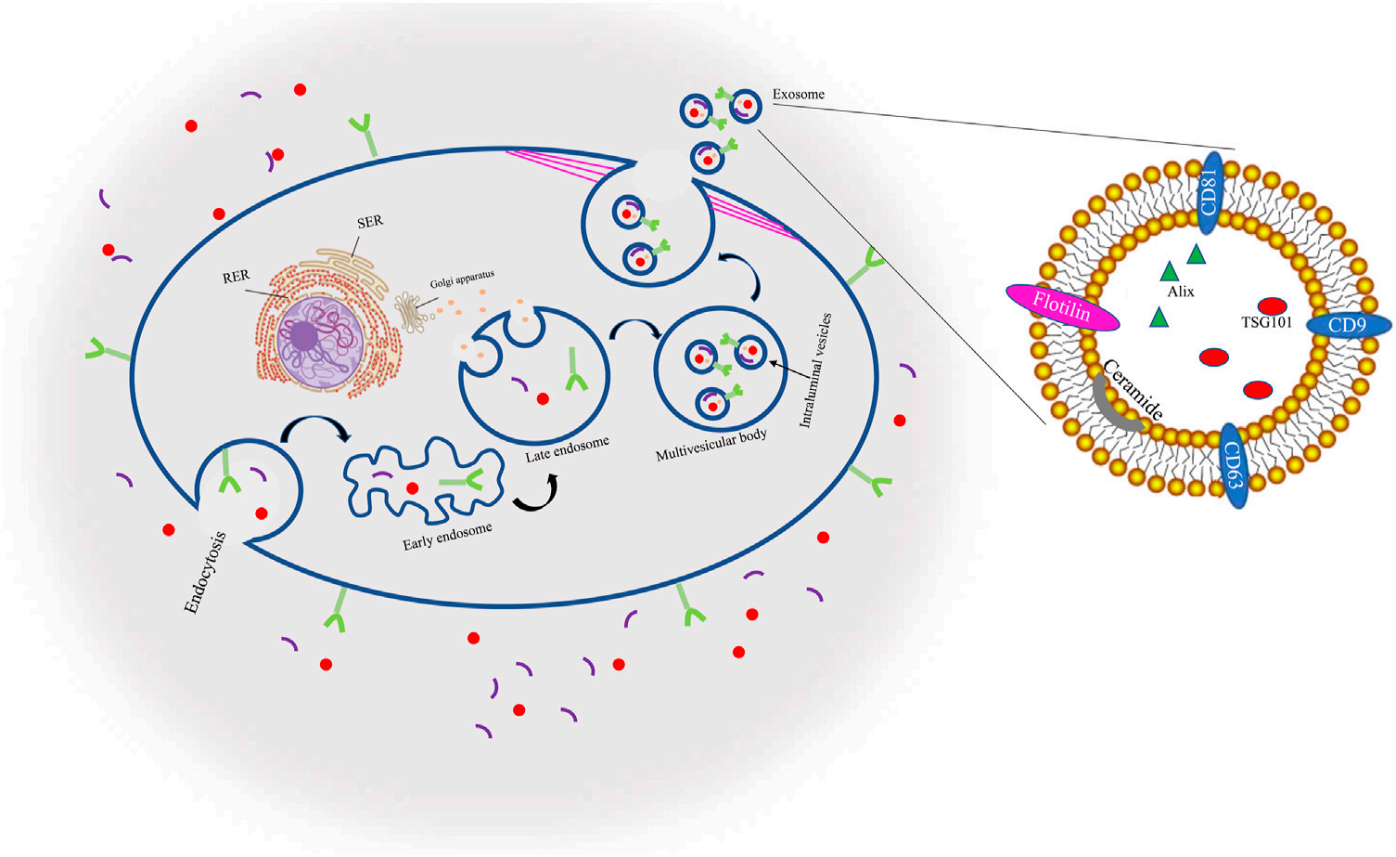
Biogenesis and identification markers of exosomes.

Through endocytosis and cell surface proteins, proteins, lipids, tiny molecules, and ions among other things can enter cells. A membrane bud then develops from the cell’s exterior to its interior, becoming what is known as an endosome. The endosome then matures, becoming a late endosome (LE) composed of closely packed intraluminal vesicles (ILVs). These vesicles are then entered by cytoplasmic components, forming what is known as multivesicular bodies (MVBs) which can either merge with autophagosomes, at which point (lysosomes destroy their contents, or they can join with lysosomes and subsequently disintegrate. Otherwise, the cytoskeleton network can move MVBs in cell microtubules to the plasma membrane where they fuse with the plasma membrane duct, a process known as exocytosis, thus forming binding proteins. Thereafter, exosomes that have a similar lipid bilayer as the plasma membrane are released (Chung et al., 2020; Rezabakhsh et al., 2021). Because of their endosomal origin, EXOs all have membrane-associated proteins that aid in EXO identification through biomarkers in classifications such as tetraspanins, heat-shock proteins, GTPases, proteins involved in forming MVBs, and antigen-presenting cells, as well as protein biomarkers such as CD9, CD81, CD63, TSG10,1, ceramide, flotillin, and Alix, Diagram1 (Mathivanan et al., 2010; Zeringer et al., 2015) (Figure 1).

## Current methods for the isolation of EXOs

Various techniques, including (ultra)centrifugation, density gradient centrifugation, commercial precipitation kits, and size exclusion chromatography (SEC), exist for use in separating MDEs; however, these techniques encounter some limitations due to the complex composition of milk.

For example, the removal of cells and cell fragments, milk fat globules, and casein micelles are challenges in these methods.

Most studies have elected to centrifuge raw milk at approximately 2,000 g to remove cells and cell debris, but no study has assessed the resulting cell or cell debris concentration. Thus, the efficacy of this method is unproven. Most research to date has obtained mixed results, because cells and/or cell fragments that survive centrifugation will co-isolate with all isolation techniques currently applied to EVs (Wijenayake et al., 2021).

During centrifugation, the fat globules in milk begin to float, forming a separate layer, which is then removed by skimming. To date, however, no study has examined the efficacy of skimming or the amount of milk fat globules left behind (Clemens et al., 2011; Yamauchi et al., 2019). The caseins present in milk create additional challenges. Casein is the most common milk protein and comprises up to 80% and 35% of the total protein in cow and human milk, respectively. Casein micelles are spherical colloidal aggregates produced by casein range from 20 nm to 600 nm in size, overlapping EVs in size. Their diameter varies with temperature; for example, human milk casein micelles measure 100 nm on average at 37°C, and 570 nm at 4°C. Casein can be removed from milk using various methods, including centrifugation, acid precipitation, calcium ion chelation, or chymosin treatment; however, to what extent the presence and functional integrity of EVs found in milk are impacted by these processes remains largely unknown (Hu et al., 2021).

### Ultracentrifugation

The gold standard among methods for isolating EXOs is ultracentrifugation (differential centrifugation). With this procedure, the biofluid is first subjected to a low-speed spin (300 g for 10 min). Dead cells and bulky apoptotic debris are likely eliminated at this speed. Then a higher speed (from 1,000 to 20,000 g)is used to remove microvesicles. Interestingly, EXOs are precipitated at the highest speed of 100,000 g (Momen-Heravi and Bala 2018). To remove the pellet and contamination or for further purification of EXOs, they can be washed in a large amount of phosphate-buffered saline (PBS) and centrifugated at a speed at 100,000 g one last time. The EXO pellets can then be resuspended in PBS and stored at −80°C for further characterization and analysis (Langer et al., 2003). Though ultracentrifugation is useful in isolating EXOs, this method is time-consuming, has low portability, and can cause damage to the EXOs because of excessive speed (Liang et al., 2017; Merchant et al., 2017; Yu et al., 2018). Therefore, differential centrifugation is not an appropriate way to isolate pure EXOs from microvesicle and apoptotic bodies because of overlapping in size with the vesicles of EXOs (Witwer et al., 2013).

By using ultracentrifugation, exosomes were isolated from both ultrasonically processed (USE) and non-ultrasonically processed (NSE) bovine milk. Marker proteins were only found in NSE by Western blot analysis. In comparison to NSE, USE had about 93% fewer microRNAs. Lipid and protein identities between NSE and USE showed a significant difference (Sukreet et al., 2019). Using conventional centrifugations and FPLC gel filtration, exosome preparations from the milk of 18 horses, were purified. The results of the protein identification were unexpected: following gel filtration, one or two peaks co-isolating proteins largely contained kappa-, beta-, and alpha-S1-caseins and their precursors, but these proteins were absent from exosomes that had been thoroughly purified. Beta-lactoglobulin, CD81, CD63 receptors, and lactadherin were present in all preparations of well-purified exosomes, although actin, butyrophilin, lactoferrin, and xanthine dehydrogenase were only discovered in part of them (Sedykh et al., 2017).

### Density gradient centrifugation

I In this method, the sample is added into an inert gradient medium for centrifugal sedimentation (Merchant et al., 2017). Various ingredients of the sample will settle on their isodensity zone under a centrifugal force, and the EXOs can then be separated from each other. One of the most important limitations in employing differential centrifugation for EXO isolation, however, is the co-precipitation of protein aggregates, apoptotic bodies, or nucleosomal fragments. The best means to tackle this problem is to use sucrose gradient centrifugation (Livshits et al., 2015). Gradient centrifugation has many benefits compared to traditional ultracentrifugation. First and foremost, this method achieves a much greater separation efficiency than the conventional method, thus providing EXOs of greater purity. Secondly, EXOs cannot be damaged and deformed with this method, and remarkably, the solution’s ingredients are prevented from remixing. Nonetheless, the instruments required for density gradient centrifugation are expensive and take up significant space in the laboratory, preventing many laboratories from acquiring them (Witwer et al., 2013).

### Microfiltration technologies

Some filtration techniques can be combined with ultracentrifugation to isolate EXOs. The ultracentrifugation technique was utilized to remove dead cells, apoptotic bodies, and large debris; then, small membranes were used for further purification. Some proteins, such as annexin V, NSE (Neuron-Specific Enolase), and PODXL (Podocalyxin), did not attach to the nanomembrane and were recovered by utilizing this technique. However, other EV proteins such as AQP2 (aquaporin 2) and *TSG101* (tumor susceptibility gene 101) connected to the nanomembrane largely could not be retrieved from the retentate. (Momen-Heravi et al., 2013). In 2010, Merchant et al. proposed a microfiltration isolation method for isolating urinary biomarkers by employing low protein-binding size exclusion filters (Merchant et al., 2010). Simply, they adopted hydrophilized polyvinylidene difluoride membranes to extract EXOs from fresh urine samples. To verify their results, they used liquid chromatography-mass spectrometry immuno-blot analysis (Cantin et al., 2008; Zhang et al., 2017).

### Antibody-coated magnetic beads

Monoclonal antibodies can attach to the surface of magnetic particles known as immunomagnetic beads with a specific target. Amazingly, the same scenario is repeatable for EXOs. Their isolation is based on the interaction between antibodies and receptor molecules on the surfaces of these vesicles (Cheruvanky et al., 2007; Merchant et al., 2010). Some receptor molecules on membrane surfaces, such as CD9, CD63, and CD81, can be utilized to isolate EXOs by employing immuno-affinity capture methods (Théry et al., 2002; Mathivanan and Simpson 2009; Tauro et al., 2012). According to this technique, an EXO magnetic complex is formed by coating these beads with antibodies against the receptor molecules of the EXOs (EXOs isolation under a magnetic field). This method is advantageous and does not require expensive instruments. Based on the expression of a specific marker and irrespective of vesicle size, a particular subpopulation can be selected and extracted from the sample. Although the majority of cells may generate a wide range of EXOs, all of them have the same markers on their surfaces. However, the antibody-coated magnetic beads technique has many limitations (Vaswani et al., 2017). Firstly, isolating EXOs from the magnetic beads is quite difficult to carry out and can result in the EXOs not able to be utilized in downstream experiments (Tauro et al., 2012). Immunoisolation-based devices have a shorter assay time (around 1.5 h)compared to other methods. Optimistic analytical tools are required to analyze EXOs when they are isolated from plasma. This method is unsuitable for point-of-care testing, so it cannot be applied to all samples. It also has expensive reagents. Furthermore, the ono-neutral PH and non-physiological salt concentrations that are adopted probably impact the EXOs’ biological activity. Last but not least, experimentation on the isolated EXOs becomes less and less possible (Lamparski et al., 2002)**.**

### Microfluidic devices

Microfluidics is the behavior and control of liquid streams that are geometrically obliged to a bit of scale at which surface powers overcome volumetric strengths. At small scales, the mechanics of fluid flow are dominated by frictional forces rather than kinetic energies. The use of microfluidic devices can be nominated as the best way to decrease material costs, energy consumption, and sample size, while also affecting growth capacity and the use of many standard laboratory processes (Le and Fan 2021). Microfluidic processes and devices can exhibit characteristic dimensions between 100 nm and several hundred micrometers, large surface-area-to-volume ratios, and low Reynolds number, holding them firmly within the laminar flow organization. One valuable technique for medical diagnoses and blood tests in clinical care is simply the use of lab-on-chip devices. Microfluidic devices are based on the binding between EXOs to the surface that can be coated by antibodies. The selected biofluid is then loaded onto a pump and injected slowly through the chip, allowing targeting isolation of EXOs (Cheruvanky et al., 2007).

### Precipitation

Utilization of an Exoquick kit is currently one of the most common strategies for extracting EXOs from human biological fluids and should be respected commercially. It is based on the law of polymer precipitation of compounds. EXOs with sizes between 60 and 180 nm are separated by mixing samples with Exoquick reagent and forming a reticulated polymer network. This strategy is faster and easier than other strategies (Chen et al., 2010), and EXOs extracted using this technique are more highly uniform in size. Furthermore, this method is the best means to isolate EXOs from small samples, for example, serum samples. However, Exoquick has a wide range of drawbacks. Contaminants with lipoprotein are likely extracted with the EXOs, which has a negative effect on analysis (Tauro et al., 2012). In addition, Exoquick is expensive and might put a significant financial strain on clinics with a high sample throughput (Merchant et al., 2017). The advantages and disadvantages of each exosome isolation method are mentioned in Table 1.

**TABLE 1:** Advantages and disadvantages of various exosome isolation methods.

Methods for the EXOs isolation	Advantages	Disadvantages	Ref
Differential centrifugation/Ultracentrifugation	Elimination of dead cells and bulky apoptotic debris in minimum (300 g) and maximum speed	Time-consuming low portability damaging to the EXOs due to excessive speed	Momen-Heravi and Bala. (2018)
	Precipitation of dead cell and bulky apoptotic debris at the highest speed (100000 g)	Overlapping with the vesicle sizes of EXOs like	
Density gradient centrifugation	High Separation efficiency	Instruments cost	Witwer et al. (2013)
	EXOs are safe	Limitation space	
	Avoiding the solution’s ingredients for mixing again		
Microfiltration technologies	Removing dead cells, apoptotic bodies, and large debris	AQP2 (Aquaporin 2) and *TSG101* (Tumor susceptibility gene 101) connected to the Nano-membrane could not be retrieved from the retentate to a great extent	Cantin et al. (2008); Zhang et al. (2017)
Antibody-coated magnetic beads	Affordable selection and extraction of a specific subpopulation from the sample can be made based on the expression of specific markers, regardless of vesicle size	EXOs isolation from the magnetic beads is so hard	Lamparski et al. (2002); Tauro et al. (2012)
		Requiring optimistic analytical tools to analyze EXOs when isolated from plasma	
		It is not suitable for point of care	
		This method cannot be applied among all Expensive reagents	
Microfluidic devices	Reducing material costs, Reducing consume energy	Large surface-area-to-volume ratios	Cheruvanky et al. (2007)
	Decreasing sample size	Low Reynolds numbers, Holding them firmly within the laminar flow organization. Suitable techniques for lab-on-chip devices	
	Growing capacity	Microfluidic devices are based on binding between EXOs to the surface can be coated by the antibody, allowing targeting isolation of EXOs	
	Many standard laboratory processes can be used		
Precipitation	Faster and easier than other strategies	Expensive method	Chen et al. (2010); Tauro et al. (2012); Merchant et al. (2017)
	Having high uniforms in their size and this way is the best means to isolate EXOs from small samples	Having high sample throughput	

## MDEs sources

**FIGURE 2:**
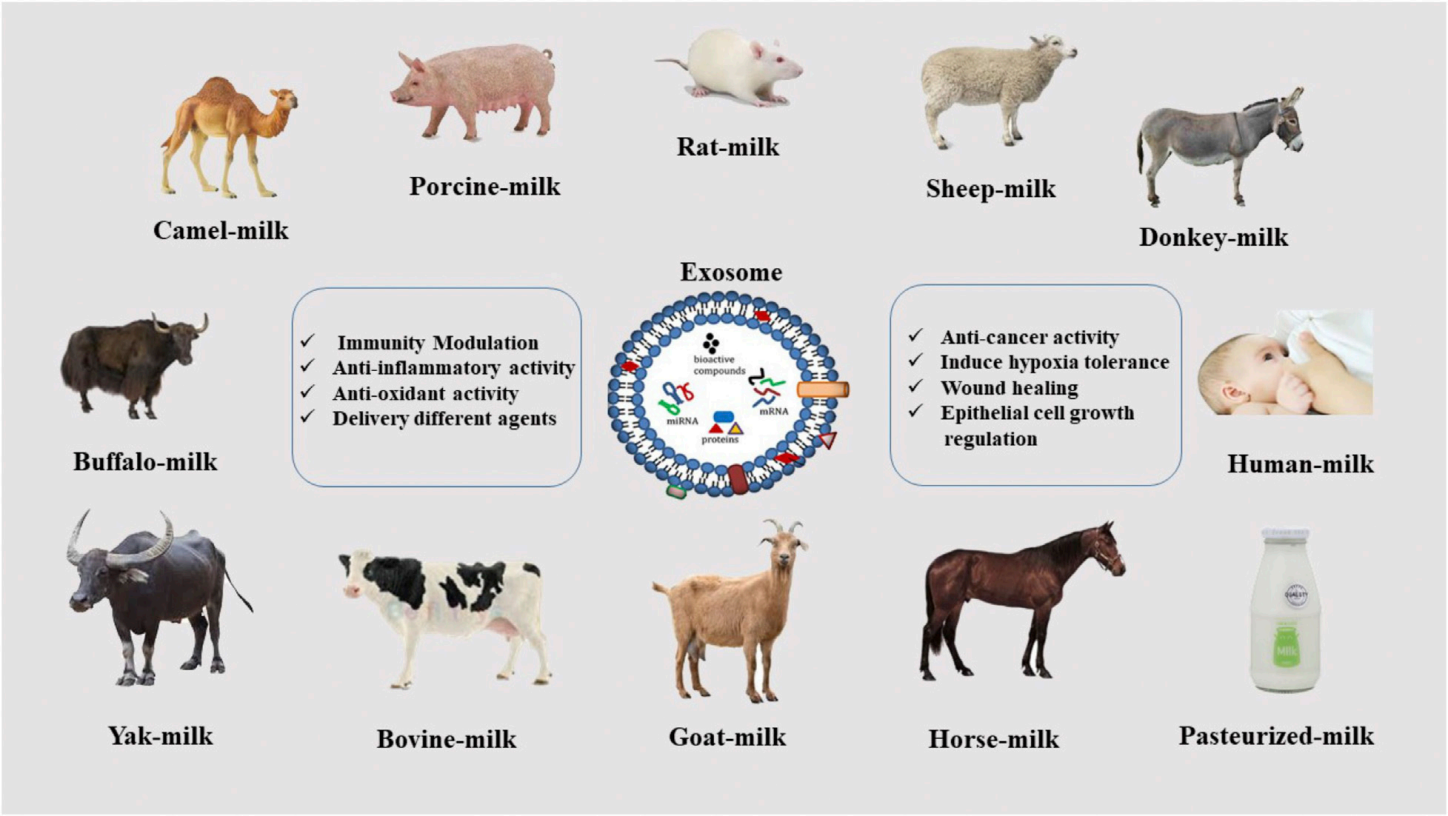
Various sources of exosome isolation from mammalian milk.

Mammalian milk is a heterogeneous fluid that contains significant amounts of biological compounds such as proteins, antibodies, and peptides. It protects infants against various diseases such as diabetes, inflammatory bowel disease, and obesity. Moreover, the antibodies in it help to strengthen the child’s immune system (Ashcroft et al., 2012). Studies have also shown that cognitive function development is better in exclusively breast-fed infants, dependent upon the duration of feeding (Shao et al., 2012). MDEs are expected to have these amazing properties, and, in fact, because they are nanoparticles, they are much easier to use in more effective medical and research processes. Studies have demonstrated the advantages of milk isolated from mammals of different species, including rat (Alvarez et al., 2012), Horse (Le Doare et al., 2018), Buffalo (Victora et al., 2016), Donkey (Hock et al., 2017), Goat (Santos-Coquillat et al., 2022), Sheep (Le Doare et al., 2018), Bovine, Human (Samuel et al., 2017; Pieters et al., 2021), Porcine (Samuel et al., 2017), Yak (Gao et al., 2019), Camel (Ibrahim et al., 2019; El-Kattawy et al., 2021). The properties of MDEs differ somewhat based on the animal species, but the most important of them are the anti-inflammatory properties, modulation of the immune system, regulation of epithelial cell growth, and antioxidant activity. A combination of these factors can be effective in the treatment of various diseases, which is discussed in the following (Ibrahim et al., 2019) (Figure 2).

## Milk–Derived exosomes applications

Milk is a source of nutrition for all newborn mammals; breast milk aids in the growth and development of the gut microbiota and immunity (Vaswani et al., 2019; Quan et al., 2020). In reference to MDEs, research has been done on drug delivery, imaging, and therapeutic applications in which MDEs can influence metabolic regulation, microRNAs function, and other biomolecules and disease treatment. These nano-carriers have been nominated as optimum for pharmaceutical ingredients (Lin et al., 2020).

Studies are currently investigating MDEs as nanodevices as novel chemotherapeutic/chemopreventive drug carriers (Feng et al., 2021). Human milk is known to induce blood clotting; a recent study demonstrated that human milk gets its coagulant activity from the tissue factor (i.e., transmembrane protein) present on EVs (Hu et al., 2020). Which appears to be the only coagulation factor present in human milk. Coagulation requires a membrane surface; because they are covered in phospholipid bilayer membranes, EVs make excellent vehicles for drug delivery. Many breast-feeding mothers suffer from nipple skin damage (Tripisciano et al., 2017; Nakamura et al., 2018). Rapid activation of the coagulation system can accelerate wound sealing, thus reducing the risk of infection. Therefore, it can be deduced that tissue factor-exposing EVs in human milk protect the mother’s health by preventing infection. Surprisingly, research has determined that this hemostasis-promoting property is totally absent in cow milk. Therefore, the lack of tissue-factor coagulant activity in bovine milk-derived EVs may signify an important functional difference in the milk of various mammalian species.

**FIGURE 3:**
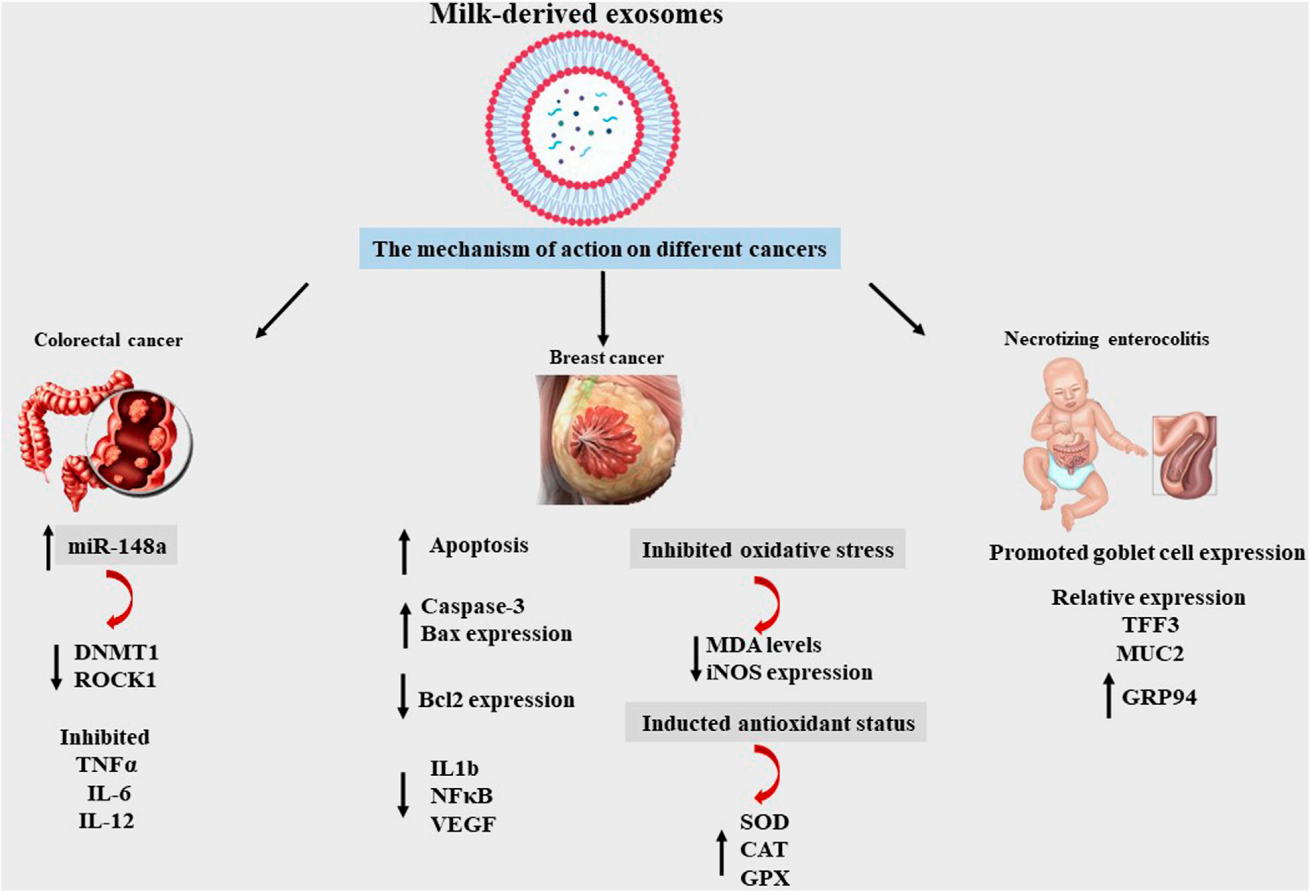
Mechanism of MDEs effects on different diseases, miRNA148a, DNA methyltransferase 1 (*DNMT1*), Rho-associated Coiled Coil-containing Protein Kinase 1 (*ROCK1*), Trefoil factor 3 (*TFF3*), Mucin 2 (*MUC2*), Glucose-regulated protein 94 (GRP94), Tumour Necrosis Factor alpha (TNF alpha), Interleukin 6 (IL6), Interleukin 12 (IL 12), BCL2-Associated X-Protein (BAX), B-Cell Leukemia/Lymphoma 2(BCL2), Interleukin 1 beta (IL1b), Nuclear Factor Kappa-light-chain-enhancer of activated B cells (*NF-κB*), Vascular Endothelial Growth Factor (VEGF), Malondialdehyde (MDA), Inducible Nitric Oxide Synthase (Inos), Superoxide Dismutase (SOD), Glutathione peroxidases (GPXs), CAT (Catalase).

Milk-derived EVs affect a breast-feeding mother’s immune responses by regulating immune cell activity. Milk-derived EVs have been reported to promote macrophage absorption of the human immunodeficiency virus (HIV)-1 while simultaneously inhibiting T cell uptake. A breast-fed infant does not contract HIV from an HIV-positive mother perhaps because dendritic cells and CD4^+^ T cells cannot acquire the HIV virus when linked to antigen-presenting cells by EVs. This type of antiviral activity against the cytomegalovirus (CMV) has been previously reported. The risk of CMV being transmitted to infants, particularly premature newborns, through their mothers’ epithelial tissue is significant; however, only a few cases of this have been reported. This may be explained by the presence of defensive mechanisms in breast milk. CMV was shown to adhere to human foreskin fibroblast-1 cells when milk EVs were processed with trypsin, suggesting that EV surface proteins may be involved (Näslund et al., 2014; Donalisio et al., 2020; Komine-Aizawa et al., 2020) (Figure 3) (Table 2, 3).

**TABLE 2:** Types of applications of MDEs in the treatment of various diseases.

MDEs origin	Study model	Major outcome	Ref
Camel	Breast cancer cells (MCF7) (*in vitro*)	Anticancer activity	Badawy et al. (2018); Ibrahim et al. (2019)
	Albino rats	Antioxidant activity	
YAK	Intestinal epithelial cell line (IEC-6) (*in vitro*)	Induce hypoxia tolerance	Gao et al. (2019)
Human	Intestinal organoids	Anti-inflammatory activity	Moatsou and Sakkas. (2019); Silva et al. (2020)
	Monocyte-Derived Dendritic Cells (MDDCs) and CD4^+^ T cells (*in vitro*)	Anti-HIV-1	
Porcine	Jejunum of a neonatal unsuckled piglet (IPEC-J2) (*in vitro*)	Intestinal cell proliferation	Chen et al. (2014)
	blood T cells (*in vitro*)	Digestive tract development and immunity of newborn piglets	Miura et al. (2022)
Bovine	Mice	Attenuates Arthritis	Ong et al. (2021); Han et al. (2022)
	Goblet cell (*in vitro*)	Rheumatoid	
		Anti-Necrotizing	
		Enterocolitis	
		Immune response and growth	
Buffalo	Bioinformatic	Preventing Infectious and inflammation	Rani et al. (2020)
Goat	Mice (*in vivo*)	Anti-inflammatory properties	Santos-Coquillat et al. (2022)
Sheep	Bioinformatic	Inflammation and immune responses during infection	Quan et al. (2020)
Rat	Intestinal epithelial cell (*in vitro*)	Anti-Necrotizing	Colombo et al. (2014)
		Enterocolitis	

**TABLE 3:** The list of Source of Milk Exosomes contains their micRNAs.

microRNA	Source of milk exosomes	REF
29b, 21,155,148a	pasteurized milk	Melnik and Schmitz (2019)
25-3p,182-5p, 200c-3p, 148a-3p	Porcine	Gu et al. (2012)
let-7a-5p,148a, let-7c	Buffalo	Chen et al. (2020)
let-7a,148a, let-7b, 21	Sheep	Quan et al. (2020)
let-7b-5p, 125a-5p,30d-5p, let-7a-5p	Human	Leiferman et al. (2019)
2,478, let-7b,1777a, 1777b	Bovine	Izumi et al. (2015)

### MDEs applications in hypoxia condition

Yaks live on the Qinghai-Tibet Plateau at altitudes of 2,500–6,000 m (Desai et al., 2021). These species have been modified in a harsh environment, resulting in adaptations to hypoxia conditions and promoted metabolic capacity. Scientific research has shown that intestinal epithelial cells (IEC-6 cell line) treated with yak-MDEs had notably higher cell survival rates under hypoxic conditions than cow-MDEs post-treatment. These findings demonstrated that yak-MDEs help improve gastrointestinal tract (GIT) development under hypoxic conditions and regulate the proliferation of IEC-6 and intestinal tract development at high altitudes through hypoxia-related pathways (Gao et al., 2019).

### MDEs applications in immune response

MDEs can transfer genetic information from a mother to her infant. This plays a crucial role in treating some diseases and results in the modulation of a newborn’s immune response. This state is likely repeated for camel-MDEs (Haug et al., 2007; Torregrosa Paredes et al., 2014). Camel milk proteins have a wide variety of benefits, such as immunomodulatory and antioxidant effects. Camel-MDEs and their related genes can improve oxidative stress and increase antioxidant properties; lastly, they can be nominated as the best EXO to regulate inflammatory patterns and improve the immune response in the cyclophosphamide (CTX)-treated species (Adriano et al., 2021; Badawy et al., 2021; Singh et al., 2021).

Human breast milk also has various components, such as milk fat globules (MFG), immune-competent cells, and soluble proteins like IgA, cytokines, and antimicrobial peptides (Zempleni et al., 2017), and can protect against early infections in infants (Pieters et al., 2015). These nanoparticles are secreted from a wide variety of cells, such as dendritic cells, macrophages, lymphocytes, epithelial, and tumor cells that belong to MHC class I- and class II-bearing nanovesicles 30–100 nm in size. They have been found in physiological fluids such as bronchoalveolar lavage, human plasma malignant effusions, and urine, and on the surface of follicular dendritic cells. The MHC class II, CD86, and the tetraspanin proteins CD63 and CD81 are expressed and exist in mammal milk and mature human breast milk, which contains EXOs (Munagala et al., 2016). Anti-CD3-induced cytokine production from peripheral blood mononuclear cells (PBMC) and increases in Foxp3 CD4^+^/CD25^+^ T regulatory cells can be inhibited because of MDEs. This suggests that the EXOs in human breast milk can influence an infant’s immune system (Admyre et al., 2007).

Porcine milk EXOs contain several miRNAs; a class of non-coding small RNAs of 18–25 nucleotides packaged in the exosomes of porcine milk may play an important role in the development of piglets. The present study revealed that these molecules greatly influenced the regulation of digestive tract development and immunity in newborn piglets. These findings increase our knowledge about the roles of miRNAs in porcine-MDEs and demonstrate the foundation for understanding their physiological functions and regulatory mechanisms (Chen et al., 2014).

The HIV-1 virus can be transferred from a mother to her child over the time period of breastfeeding, however the percentage of transmission possibility is less than 30%. Studies have shown a lower risk of postnatal HIV-1 infection in exclusively breastfed infants than in mixed breastfed children during the first months of life. Many studies have shown that components in milk, such as bile-salt stimulated lipase (BSSL) and soluble mucin 1 (MUC1), can provide barriers to protect dendritic cells against HIV-1 infection (Näslund et al., 2014).

### MDEs applications in intestinal diseases

Necrotizing enterocolitis (NEC) is one of the most common intestinal diseases and has a high rate of mortality in premature and fragile infants. The symptoms of this disease range from colonic inflammation to intestinal perforation, extensive necrosis, multiple organ failure, and death. Bovine-MDEs can prevent intestinal injury by increasing the number of goblet cell and ER (endoplasmic reticulum) functions and have been shown to impact NEC prevention in experimental mice by improving mucin expression by goblet cells. In the inflamed intestine, depletion of mucin production from goblet cells occurs prior to epithelial cell damage. Further studies have demonstrated that milk-derived EXOs decrease myeloperoxidase (MPO) expression in experimental NEC. Interestingly, the beneficial anti-inflammatory effect of EXOs is associated with the restoration of mucin production (Li et al., 2019).

Milk-derived EXOs contribute to reducing colitis induced by dextran sulfate sodium (DSS) and histopathological scoring grade, and statistics have shown that shortening of the colon can be reduced. Moreover, the expression of interleukin 6 and tumor necrosis factor-alpha can be reduced by treatment with MDEs. Furthermore, miRNAs such as miRNA-320, 375, and Let-7 are highly expressed in milk and can be found in the colon of MDE-treated mice compared with untreated mice. It has been indicated that miRNAs play an important role in treating colitis by using MDEs and regulating the expression of target genes (Reif et al., 2020).

Certain studies have suggested that colorectal cancer cells can be reduced by utilizing exosomes obtained from pasteurized milk. The pMDEs have two primary micRNAs, micRNA148a and micRNA155, and the expression levels of these two genes—DNA methyltransferase 1 (*DNMT1*) and rho-associated coiled coil-containing protein kinase 1—were reduced (*ROCK 1*). Both genes were blocked by both micRNAs, because they can cause tumor progression and metastasis. Moreover, micRNA 148a is essential to controlling the immune system, as it reduces cytokines such as TNF, IL6, and IL12, among others (Melnik and Schmitz 2019).

### MDEs applications in cancer therapy

Previous studies have demonstrated the anticancer effects of crab blood-derived exomes. Recent research has shown that milk, as a biological fluid, also has anticancer properties (Othman et al., 2021; Rezakhani et al., 2021; Rezakhani et al., 2022). Camel-MDEs have a wide range of benefits, such as reducing metastasis of breast cancer, 2) increasing the number of markers of apoptosis, and 3) reducing oxidative stress and gene expression related to inflammatory and immune response induction (Badawy et al., 2018; Abu-Farha et al., 2020). Another study has shown how cow milk can provide notable amounts of exosomes, which can transport chemotherapy and chemoprevention medications. Drug-loaded exosomes have been shown to be significantly more effective than free exosomes in preventing lung tumor xenografts *in vivo* and in cell culture tests. Tumor-targeted ligands such as folate have also been reported to enhance the targeting of exosomes by cancer cells, thereby intensifying tumor reduction (Munagala et al., 2016).

Chemotherapeutic agents such as paclitaxel can be loaded into EXOs in the membrane’s lipid bilayer. Exosomes can carry stable drugs in simulated gastrointestinal conditions and are suitable carriers for oral drug delivery. These nanoparticles can be adopted to load vast amounts of curcumin as an optimum route against tumors. Exosomes carrying curcumin can address some challenges associated with curcumin, such as lack of stability, solubility, and bioavailability in the adverse conditions of the digestive tract compared to free curcumin (Strobel 2001; Rappa, Caruso Bavisotto et al., 2019; Corrado et al., 2021).

Few studies have investigated exosomal drug encapsulation for the oral delivery of peptide/protein medications, and what biological factors underpin their capacity as oral delivery vehicles remain unknown. Insulin-loaded milk-derived exosomes (EXO@INS) have been developed, and their hypoglycemic effects were examined *in vivo* in type I diabetic rats. Surprisingly, EXO@INS achieved a greater and longer-lasting hypoglycemic effect than that of subcutaneously administered insulin (Wu et al., 2022).

As indicated above, yak-MDEs reduce hypoxic conditions, a key point in treating cancer cells, because there is much less tumor oxygen in the microenvironment than in another environments, which explains why metastasis can occur easily. Yak milk EXOs, however, can overcome these conditions and improve the treatment of cancer cells (Gao et al., 2019).

### MDEs applications in wound healing

EXOs are biocompatible and produced by natural cells; they control the inflammatory response and promote cell migration and proliferation, which makes them one of the best treatments for wound healing. All of the positive aspects of EXOs occur because of their compounds. Stem cells have been most commonly used for wound healing until now; however, aspects of stem cell usage, including biosafety, administration, and bio-distribution, should be more deeply investigated, and an alternative in more bio-stable materials is needed (Yan et al., 2022). The mechanisms of scar-free healing are not yet clearly understood. Apparently, TGF-b3/TGF-b1 is a key factor in the wound healing process. Research has shown that MDEs in Intestinal Epithelioid Cell line number 18 (IEC-18 cells) induce anti-cell migration. In addition, the expression of transforming growth factor beta-3 (TGFb3) was shown to be elevated in response to Mi-EXO treatment, but the level of TGFb1 remained unchanged Research has indicated that wound healing primarily follows the TGFb/Smad signaling pathway. Smad protein is a crucial transcription factor in TGFb signaling that has a different function than TGFb, including 1) receptor-activated Smad (Smad1, Smad2, Smad3, Smad5, and Smad8); 2) common mediator Smad (Smad4); and 3) inhibitory Smad (Smad6 and Smad7). Smad3 protein is phosphorylated because of the activation of transforming growth factor-beta receptor1 (TGFbRI) and transforming growth factor-beta receptor 2 (TGFbRII), and phosphorylated Smad3 plays a significant role in cell growth and ECM formation. Generally, these findings suggest that MDEs could be an interesting material for minimizing various scars or keloids, such as skin tissue damage, abrasion, acne extrusion, and surgical skin incision (Ahn et al., 2021).

**FIGURE 4:**
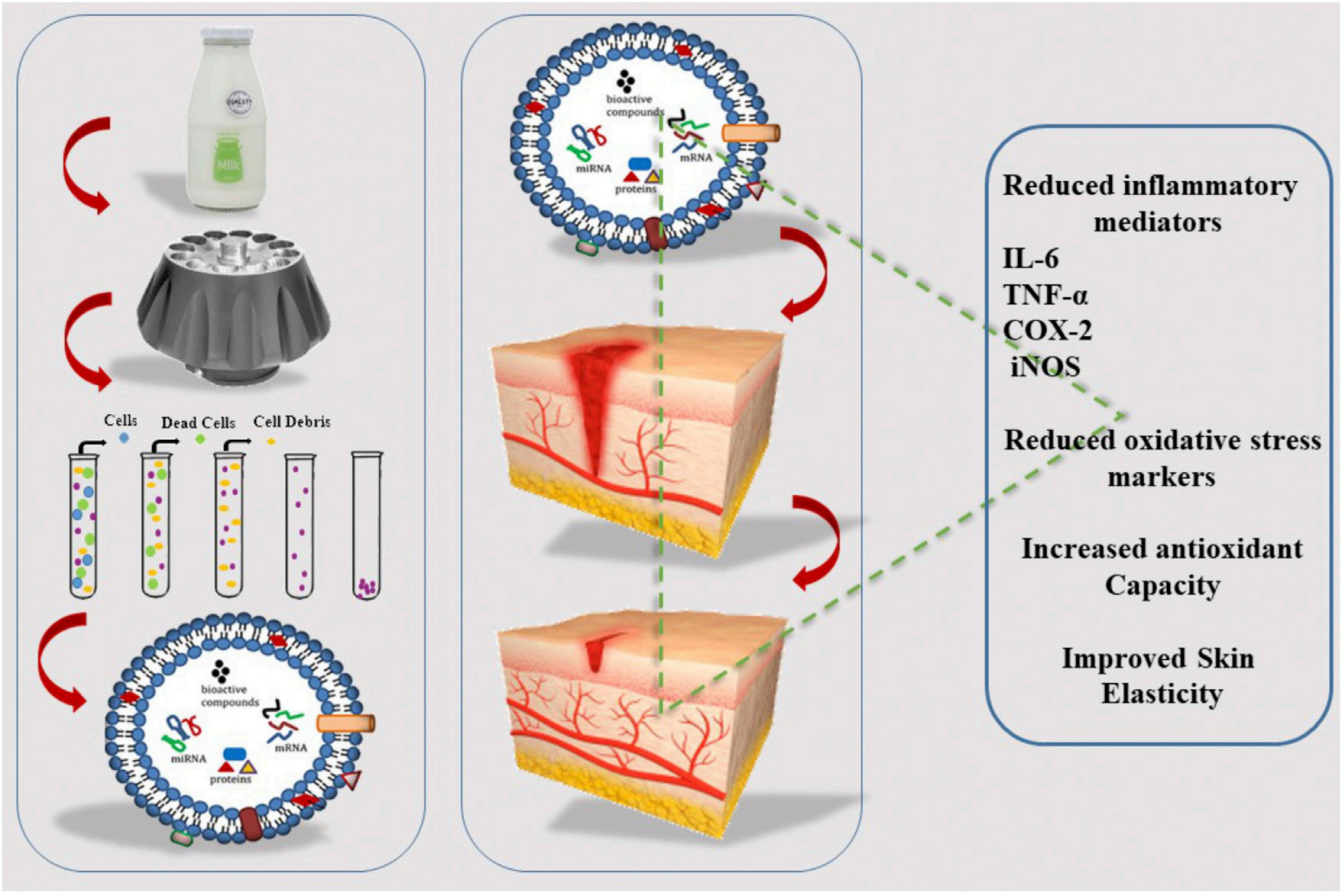
Isolation of EXOs from milk and its effect on wound healing, cyclo-oxygenase (*COX-2*), Tumor susceptibility gene 101 (*TSG101*).

Research has shown that bovine milk-derived EXOs positively impact UV-induced aging and damage in keratinocytes, melanocytes, and fibroblasts put in three parts of skin cells. Interestingly, milk EXOs can prevent the induction of UV and intracellular reactive oxygen species in epidermal keratinocytes. In UV-stimulated melanocytes, milk EXOs can reduce the production of the skin-darkening pigment melanin, which may reduce the production of vast amounts of melanin caused by skin hyperpigmentation disorders. Milk EXOs can suppress the expression of matrix metalloproteinase in human dermal fibroblasts. In contrast, increased cell proliferation was accompanied by enhanced production of collagen, a major extracellular matrix component of skin. Remarkably, research indicates that bovine milk-derived EXOs have great potential as natural therapeutic agents to repair UV-irradiated skin aging and damage (Han et al., 2022) (Figure 4).

### The applications of MDEs in Bone-related diseases

The bioactive ingredients found in milk significantly influence bone metabolism. Research has shown that proteins isolated from bovine milk can reduce bone loss. These nanoparticles have many microRNAs, which have beneficial impacts on the host. The effects of bovine milk–derived EXOs (BCE) on osteoclast differentiation has been evaluated *in vitro* conditions, and the results indicate that BCE is beneficial for osteoporosis in an animal model (Vashisht et al., 2017; Li et al., 2019; Raimondo et al., 2019).

## Challeng of exosomes in clinical medicine

Harvested EXOs lack sufficient conventional surface markers but contain other extracellular vesicle types, such as microvesicles; therefore, they usually suffer from co-isolation and impurities. When employing conventional techniques like ultracentrifugation, one must be acutely aware of morphological and functional changes in EXOs. Exosomes isolated using high-speed pelleting can incur mechanical damage, protein aggregation, lipoprotein contamination, and low-rate purity, and using ultracentrifugation may result in low yield rates and exosomal payloads. Extracting EXOs by ultracentrifugation may result in some indicators having different final concentrations compared to parent cells. Previous studies have shown that extracting EXOs through ultracentrifugation lowered calnexin but left CD81 and CD9 levels unchanged. Storage presents a significant issue when applying EXOs in regenerative medicine. It has been suggested that the lack of storage options may cause modifications to their size and composition. In temperatures of 4 and 20°C, EXOs showed more severe changes compared to lower temperatures like 80°C. For example, CD63 and HSP70 levels were reduced when EXO was kept for 10 days at higher temperatures, such as 4°C. Notably, exosomal cargo loss was greater at room temperature. The dispersion of XOs became more even as storage conditions became warmer. Phosphate-buffered saline is commonly used as a cryopreservation storage buffer in procedures. Trehalose is a substance that can be added to phosphate-buffered saline to avoid EXO edema. Cryodamage (i.e., exosomal aggregation) occurs when objects are kept at low temperatures and results in the loss of EXO functionality after administration. EXO aggregation and subcellular localization is affected by the number of freezing/thawing cycles after treatment with target cells. Studies have shown the absorption of EXOs by cells and storage pH. EXOs kept at a pH between 4 and 10 displayed higher uptake levels than those maintained at pH 7. Further studies are necessary to complement our understanding of the underlying processes that provide optimal cryopreservation without compromising exosomal integrity and function (Haraszti et al., 2018).

## Conclusion

The promising results of research in recent years on the use of EXOs as drug carriers and biomarkers have been of great interest to scientists. MDEs have been introduced to the scientific community as biocompatible and cost-effective nanoparticles with high availability and the potential for preparation in high volumes. Considering the unique properties of these nanoparticles, they can be considered suitable candidates for use in treating diseases. Further study of different approaches and treatment strategies with MDEs are suggested to determine appropriate future cure plans for various diseases.
